# Supplementary figures and images for: Addition of Combinedly Dehydrated Peach to the Cookies—Technological Quality Testing and Optimization
Source: Foods. 2022 Apr 27;11(9):1258. doi: 10.3390/foods11091258 (PMC9099911; doi:10.3390/foods11091258)

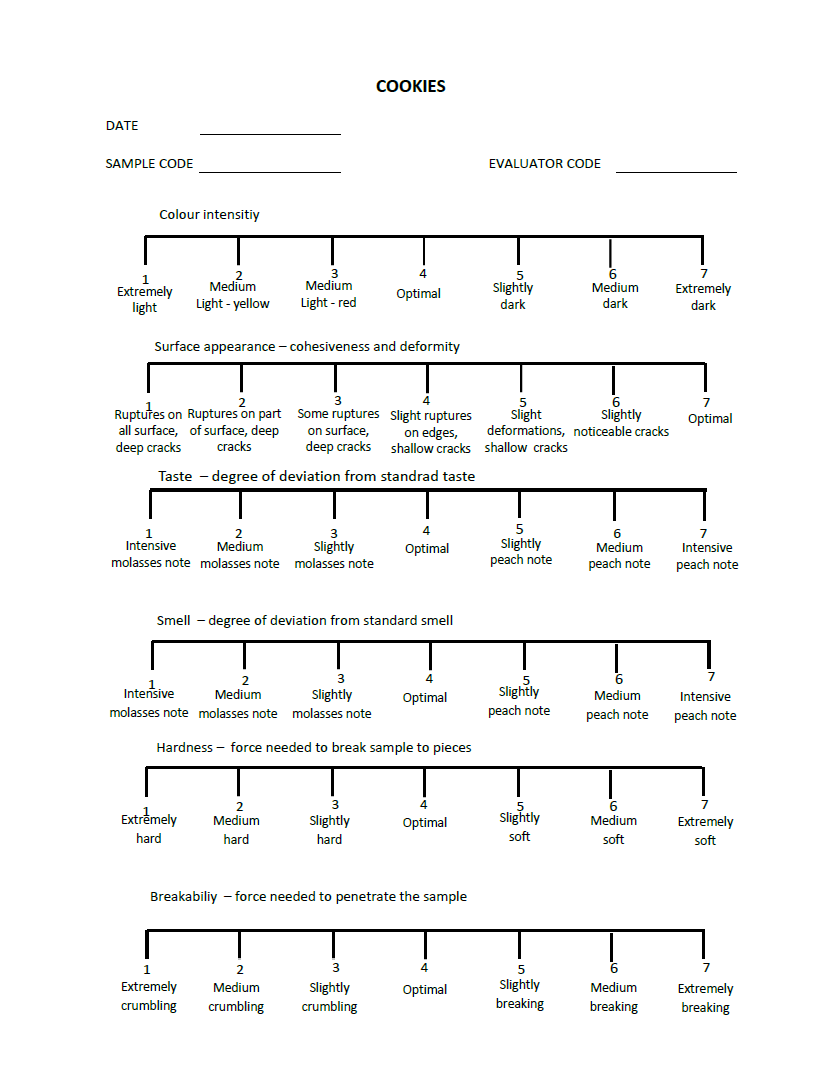

Supplement: Supplementary file 1 [file foods-11-01258-s001.zip › foods-1654935-supplementary.tif]
